# Tracking pregnant women displacements in Sao Paulo, Brazil: a complex systems approach to regionalization through the emergence of patterns

**DOI:** 10.1186/s12916-019-1416-4

**Published:** 2019-10-01

**Authors:** Felix Rigoli, Sergio Mascarenhas, Domingos Alves, Tiago Canelas, Geraldo Duarte

**Affiliations:** 1 School for International Training, São Paulo, Brazil; 2 Institute of Physics, University of Sao Paulo, Sao Carlos Campus, Brazil; 3 Department of Social Medicine, Ribeirão Preto Medical School, University of São Paulo, Ribeirão Preto, Brazil; 4 Department of Vector Biology, Liverpool School of Tropical Medicine, Liverpool, UK; 5 Department of Gynecology and Obstetrics of Ribeirão Preto Medical School, University of Sao Paulo, Sao Carlos Campus, Brazil

**Keywords:** Complexity, Maternal-child health services, Regional health planning, Public health systems research, Unified health system

## Abstract

**Background:**

The healthcare system can be understood as the dynamic result of the interaction of hospitals, patients, providers, and government configuring a complex network of reciprocal influences. In order to better understand such a complex system, the analysis must include characteristics that are feasible to be studied in order to redesign its functioning. The analysis of the emergent patterns of pregnant women flows crossing municipal borders for birth-related hospitalizations in a region of São Paulo, Brazil, allowed to examine the functionality of the regional division in the state using a complex systems approach and to propose answers to the dilemma of concentration vs. distribution of maternal care regional services in the context of the Brazilian Unified Health System (SUS).

**Methods:**

Cross-sectional research of the areas of influence of hospitals using spatial interaction methods, recording the points of origin and destination of the patients and exploring the emergent patterns of displacement.

**Results:**

The resulting functional region is broader than the limits established in the legal provisions, verifying that 85% of patients move to hospitals with high technology to perform normal deliveries and cesarean sections. The region has high independence rates and behaves as a “service exporter.” Patients going to centrally located hospitals travel twice as long as patients who receive care in other municipalities even when the patients’ conditions do not demand technologically sophisticated services. The effects of regulation and the agents’ preferences reinforce the tendency to refer patients to centrally located hospitals.

**Conclusions:**

Displacement of patients during delivery may affect indicators of maternal and perinatal health. The emergent pattern of movements allowed examining the contradiction between wider deployments of services versus concentration of highly specialized resources in a few places. The study shows the potential of this type of analysis applied to other type of patients’ flows, such as cancer or specialized surgery, as tools to guide the regionalization of the Brazilian Health System.

## Background

The health system in a country is usually conceptualized as a systematic arrangement of hospitals, clinics, and other providers, following a neat pattern of primary care facilities that refer patients to a more equipped hospital, as first proposed in 1920 by the Dawson Report [1]. However, the actions of patients, providers, and government configure a much more complex network of interactions. It should be noted that this article restricts the use of the term “complex” and “complexity” to the features of the health system that may be analyzed using a complex systems approach [2] avoiding the colloquial use of complexity as a byword for not easy to understand, complicated or intricate [3].

In order to better understand such a complex system, the analysis must include characteristics that are feasible to be studied in order to redesign or reorient its functioning. As per Mitchell [2], one way of studying the functioning of a complex system is to explain how large-scale complex adaptive behaviors emerge, as may be seen in diverse examples such as the way coherence rises from chaotic neuron firings or the order coming out from a myriad of individual movements in the cities. Castellani et al. [4] described nine essential complex system characteristics related to the relations of territory and health: (1) causally complex, (2) self-organizing and emergent, (3) nodes within a larger network, (4) dynamic and evolving, (5) nonlinear, (6) historical, (7) open-ended with fuzzy boundaries, (8) critically conflicted and negotiated, and (9) agent-based. Bar-Yam [5] attributes the problems faced by health system planners to the existence of a fine-scale complex system (the countless variations created by individual needs and providers availability), confronted with a large-scale non-complex deterministic system encompassing the legal and economic infrastructure of budget and staff. Accordingly, the pretension of economists of determining fixed parameters for healthcare supply and demand, as well as trying to use incentives to guide the behavior of communities and practitioners, does not take into account the possibility of better solutions emerging from the agents’ interactions [6].

In the study of complex health systems, the emergence of patterns can be used as a leading thread to understand the underlying dynamics that force adaptations to the system. The usual approach of breaking down complex interactions to its components [7], in order to act in separate factors and outcomes, conceived as causes and effects, fails to recognize the connections and feedback loops among the parts, conducing to failures interpreted as “policy resistance” [8] or “turbulence” [5].

The analysis of the patterns of patient flows using a complex adaptive systems approach can provide support to understand the wider scope of the health organization dynamics [9–13]. The study may influence the ongoing process of defining the Brazilian Unified Health System (SUS) regional configuration, a problem that has puzzled planners since the creation of the SUS, meriting six different layouts of regional boundaries since 1990 [14] as the flows of people dynamically shape the configuration of territories [15, 16]. The self-organizing behaviors emerging from the interactions of individuals with each other and their territories are not easily subject of traditional linear analysis and planning, demanding new models to be applied. As suggested by Auchincloss and Diez Roux [17], “These limitations have constrained the types of questions asked, the answers received, and the hypotheses and theoretical explanations that are developed. [ …] Using these (agent-based) models, one can observe how macroscale dynamics emerge from microscale interactions and adaptations”. At the same time, they offer tools to explain why and how the organization of the system needs to be adapted to larger environment changes.

### Relevant features of the Brazilian health system

Brazil has developed its constitutional mandate to provide universal right to health by drawing together several previous public health systems within the Unified Health System (SUS). The SUS provides universal coverage in a country of 208 million people and more than 8 million sq. km. The challenge of delivering health care while guaranteeing equal access in such a diverse country leads to a service structure divided according to state and municipal authorities. By its constitutional organization, it is a federal country with 27 states and 5561 municipalities that range from less than 1000 to more than 12 million inhabitants. More than 70% of those municipalities have less than 20,000 inhabitants. Due to the diversity of regions, resources, and other characteristics, the system is financed by a variety of arrangements of federal, state, and municipal funds. These funds are channeled towards the network of services through several legal and budgetary arrangements that combine funding for hospitals, primary care teams, and preventive programs. As these funds are mostly proportionate to the state and municipal population, there are important imbalances in the amount of funding that can be gathered in each municipality, and thus, the level of services that each territorial unit can provide is heterogeneous. Most states are larger than many countries, and not able to be managed as a unit. On the other hand, the majority of municipalities are too small to provide anything that exceeds the basic health services.

This imbalance in scales is being confronted through grouping municipalities in health regions. The difficulties in associating different levels of political units in order to create arrangements among such diverse array of municipalities have resulted in a mismatch between needs and service availability, that are especially acute in regions that encompass rural and urban areas, as well as considerable geographic distances. This discrepancy between needs and availability is a factor underlying the successive remodeling of the health regions [14].

### Patients’ flows patterns and health system organization

In order to improve the regional organization, studies of flows of SUS users may be indicative of self-organizing adaptive behaviors, in turn pointing to better ways of designing and rearranging this regional organization and guiding the regional planning of resources [16].

The problem that was selected to explore these patterns refers to the regional distribution of facilities for safe childbirth and has as its inevitable counterpart the displacements of patients. This problem confronts two contradictory elements: the wider distribution of care centers vs. the high quality of resources needed for institutional care of the childbirth. These two aspects will be described separately and then combined to define the problem to be researched.

Most countries accept that institutional delivery is a cornerstone of a good functioning health system, determining different health outcomes of a population, especially maternal mortality and child mortality. For that reason, it was included as the Indicator 5.2 in the Millennium Development Goals [18] also contributing to the Sustainable Development Goal 3, targets 1 and 2 [19].

The regional distribution of human and technological resources devoted to solve a specific problem such as the resolution of pregnancy is at the center of the dilemma between equity and efficiency that every universal health system must face. This dilemma does not preclude the search for practices that are effective and appropriate, as pre-conditions to make a procedure available for all. On the one hand, services with a high concentration of resources and expertise, located in central places [20], guarantee high quality and efficiency [21, 22]. On the other hand, this concentration may make it difficult for patients from remote regions to access those services. WHO distinguishes two levels of obstetric care, basic and comprehensive [23], and proposes that countries should provide the highest possible level. SUS regulations address this potential conflict by ensuring that all citizens have access to services including “prenatal care, childbirth, and the puerperium” as closely as possible to their residence [24].

The approach to resource allocation dilemmas for health may be described under the term “wicked problems” [25] that have contradictory solutions, depending on the point of view or the stakeholders’ interests. The flow of patients that need to move away from their municipality of residence in order to have their delivery-related hospitalizations is therefore an expression of this type of problem. If viewed from the standpoint of easiness of access, the optimal solution would be guided by the wide distribution of maternity centers in the municipalities; on the other hand when considered the quality of care and potential life-threatening risks, the tendency should be to have a small set of well-equipped centers with high-level trained staff. Traditionally, the power imbalance between the clinicians’ time and the patients’ rights forced the latter to travel more to search for assistance [26], but this should not be acceptable within the approach of a patient-centered universal health system. To mitigate this problem, the state of São Paulo health planners use an algorithm to refer patients that show up at municipal emergency rooms in order to reach the best technically equipped public facility, even when this hospital is without available beds [27].

**Fig. 1:**
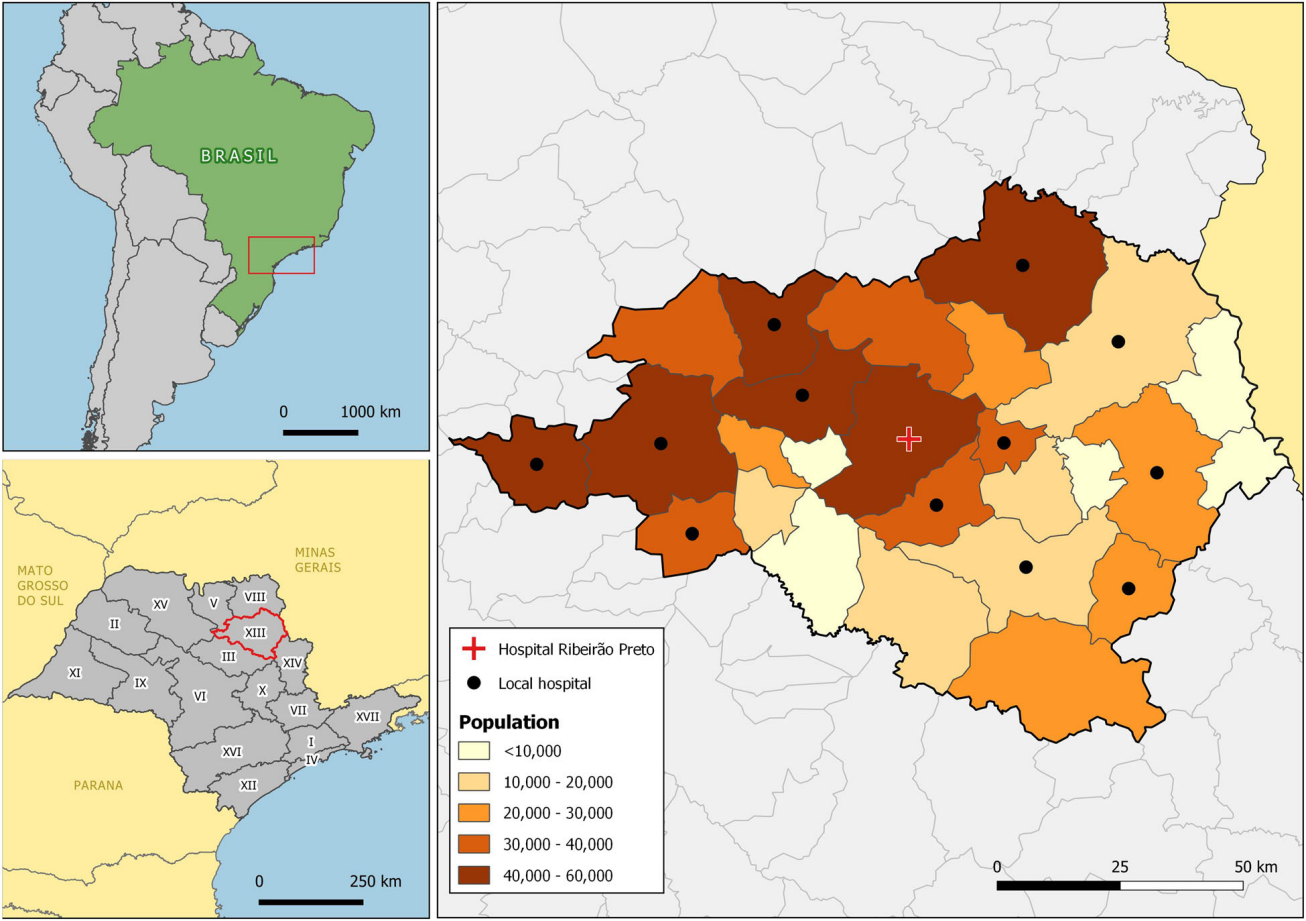
Regional Health Department XIII within São Paulo state and Brazil. Legends: In green: Brazil in South America with a square showing the São Paulo region. In gray: São Paulo state health regions. Highlighted in red, the Regional Health Department XIII centered in Ribeirão Preto. Source: [28, 29]

The rationale for this research is to describe the emerging patterns of patient flows for the resolution of pregnancy in a region of São Paulo, Brazil, centered in the Regional Health Department XIII (DRS XIII) (Fig. 1), providing a new kind of information that could support a series of interventions and strategies for the flows of pregnant women in this region and ultimately to improve the functionality of health regions. The issue is particularly important because it is a crucial event for the survival of the dyad mother/child, and is an event that should happen as closely as possible to the municipality of residence, according to existing regulations. In this sense, every cross-border flow may be considered an anomaly. On the other hand, the emerging patterns can also provide information to help resolve the regional resource distribution dilemmas for this clinical situation and create a blueprint for other health conditions.

## Methods

The research uses spatial interaction models of patients’ inter-municipal flows for the resolution of pregnancy, operationally defined as patients admitted under code XV of ICD 10 in hospitals belonging to the administrative region surrounding Ribeirão Preto, Brazil, during 2012.

The study is based on the analysis of the areas of influence of the hospitals of Ribeirão Preto for pregnancy-related cases taking into account the displacements between points of origin and destination of the patients. Spatial interactions models such as migration and transportation are based on a location-to-location network (graph) in which a node represents a location and a link (arc or edge) represents an interaction (flow) between two locations.

The information sources for this research came from the Regional Health Department XIII (DRS XIII) centered in the city of Ribeirão Preto, the obstetrical referral service of the Department of Gynecology and Obstetrics of the Ribeirão Preto Medical School of the University of São Paulo (DGO-FMRPUSP), and the Regional Observatory for Hospital Care – ORAH [30]. The ORAH works with 36 hospitals located in DRS-XIII and tracks 170,000 hospital admissions per year [31–34]. The DGO-FMRPUSP houses the regional research in maternal health within its Postgraduate Program, Doctoral Level, and serves as a reference center for the attention of high-risk maternal or perinatal cases in the region. For this reason, it is a privileged vantage point to understand the details of the operation (and the dysfunctions) of the primary and secondary care network.

The DRS XIII is composed of 26 contiguous municipalities and is part of a regionalized health structure in the State of São Paulo. Internally, it is subdivided into three micro-regions, namely, Vale das Cachoeiras (VdC), Horizonte Verde (HV), and Aquifero Guarani (AG). These three micro-regions are supposed to have some administrative independence in terms of solving their health cases, even though there are no legal or administrative rules regarding their list of competences. Due to the influence of Ribeirão Preto as a center for services, much of the micro-regional activity is referred to the main city.

The study encompasses all cases in the ORAH database of DRS XIII for 2012. This was the last year available with more complete and clean data for the whole region.

The analysis was based on an origin-destination matrix of those municipalities that send out more than 5 patients [35] to be admitted in any of the 36 hospitals of the DRS XIII in 2012. The flows contained in the origin-destination matrix were aggregates of the patients residing in municipality *i*, hospitalized for procedures related to their pregnancy, delivery, or puerperium in the municipality *j*. The Σij for each pair of municipalities that experienced flow of patients is a directed edge or arc. Directed edges express non-symmetrical relations, in this case, a flow from residence to hospital.

For the intensity calculations of the flows, we chose to use the intensity in the municipality of origin (i.e., the proportion of cases that migrate). Network analysis was applied to patient flows [36]. The software used was *UCINET®* [37] and QGIS (*ver2.14 GNU General Public License*)®.

Based on these displacements, the research mapped out areas of influence of each of the municipalities that receive hospitalizations for pregnancy, childbirth, and puerperium, analyzing different measures and thresholds for estimating regional influence.

The network analysis allowed to visualize main network features and subnets created by these flows and comparisons with the subnets defined by the regulations.

The distance-decay from a central place or gravitational effect [38] was calculated using the coefficients LIFO (little in from outside) and LOFI (little out from inside) first described by Elzinga and Hogarty [39, 40] and later applied to health markets [41, 42] in order to determine the sufficiency or independence of the municipalities and regions. The expression used is:

LIFO = 1 − (patients received from outside the municipality/total patients hospitalized in the municipality) expressed in percentage.

LOFI = 1 − (patients leaving the city of residence to be hospitalized/patients residing in the municipality receiving hospitalization) expressed in percentage.

Most studies use LOFI and LIFO levels between 75 and 90% [36–39] in order to delimit an area of influence of the flows. According to this methodology, concomitant levels of the two parameters are necessary to determine if a given area is sufficient enough to treat its patients as well as not critically needed by their surrounding areas. For the purposes of this research, the sufficiency or independence of a region was defined by pairs of LOFI/LIFO values higher than 75%. As originally formulated, “a region that is successfully defined on an ecological basis will have intraregional interactions, which are quantitatively and, in the most desirable case, qualitatively distinguishable from interregional interactions” [43].

According to the additional method proposed by Frech et al. [42], the delimitation of the sufficiency area was evaluated extending the number of geographical units until the set proved to be sufficient by the paired LIFO/LOFI criteria.

## Results

**Fig. 2:**
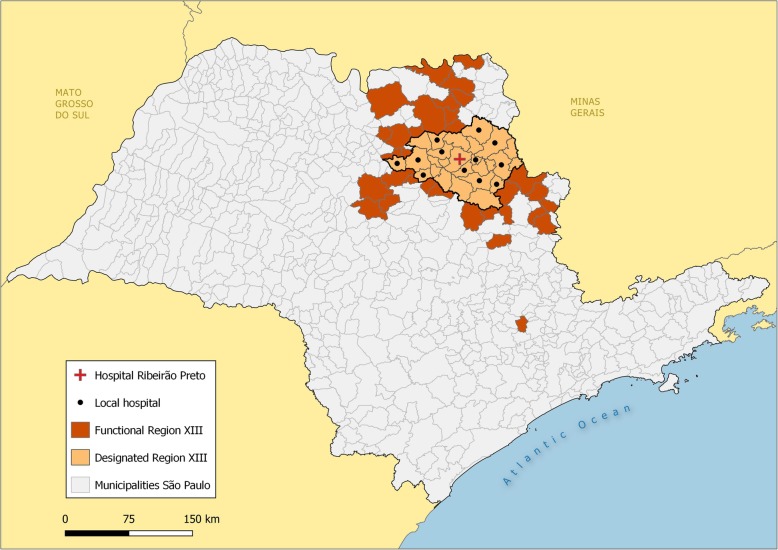
Comparison between designated and functional regions for deliveries, São Paulo state, 2012. Legends: São Paulo state municipalities. In orange, municipalities in the designated Health Department XIII. In red, municipalities in the functional region derived from data. Source: [28, 29]

The DRS XIII (Fig. 1) is legally composed by 26 municipalities; however, the studied region ended up encompassing 60 municipalities that had significant (> 5) flows of patients for admissions related to pregnancy, childbirth, and puerperium in DRS XIII hospitals (Fig. 2).

These 60 municipalities present a relatively homogeneous demographics: high life expectancy (average 75.5 years), low fecundity rate (1.89), and an average high Municipal Human Development Index of 0.745 with a standard deviation of 0.03, revealing quite uniform social conditions.

An analysis of the flows of events related to childbirth and puerperium in 2012 reveals that in the municipalities of DRS XIII, there were 19,834 hospitalizations due to pregnancy, childbirth, and the puerperium, of which 5043 were originated in patients admitted in a different municipality. In principle, these hospitalizations denote some exceptional circumstances, since the delivery care should be the responsibility of the municipality of residence or at least of the micro-region or health region. In the case of the municipality of Ribeirão Preto, 85% of the hospitalizations coming from outside DRS XIII were concentrated in two categories: normal delivery and non-complicated cesarean delivery that are not a priori exceptional circumstances. In spite of the positive aspects of hospital availability, its widespread use is also pushing up the rate of unnecessary procedures as C-sections, a trend that is being observed across Brazil. The Ribeirão Preto hospitals had in 2012 74% of C-sections, well over the 2010 national average of 44% [28] and the São Paulo state average of 58% for 2009 [29].

In the studied region, there are 36 hospitals with a total of 3278 beds of common hospitalization, thus offering an availability of 2.46 beds per thousand inhabitants. Ribeirão Preto is an important city in São Paulo state, concentrating four medical schools and the main centers for clinical excellence, gathering the best-equipped facilities and the most qualified teams in the region. The municipality offers 75% of the total hospital capacity for a population that is 52% of the total. Patients and technical staff acknowledge this regional qualitative and quantitative excellence in the hospital availability, contributing to the centrality of Ribeirão Preto for attracting medical procedures.

The research plotted the municipalities of origin of the patients, totaling 5043 cross-border admissions (25.4% of the total). The mapping of the municipalities of origin showed that the functional area goes beyond the designated limits of DRS XIII for pregnancy, childbirth, and puerperium-related hospitalizations (Fig. 2). The functional region comprises more than 30,000 km^2^ and distances of 160 to 200 km in its main axes.

The observed average distance of displacement of the patients who left their municipality to go to Ribeirão Preto was 47.56 km, while the average of distance for the rest of patients was 27 km.

**Fig. 3:**
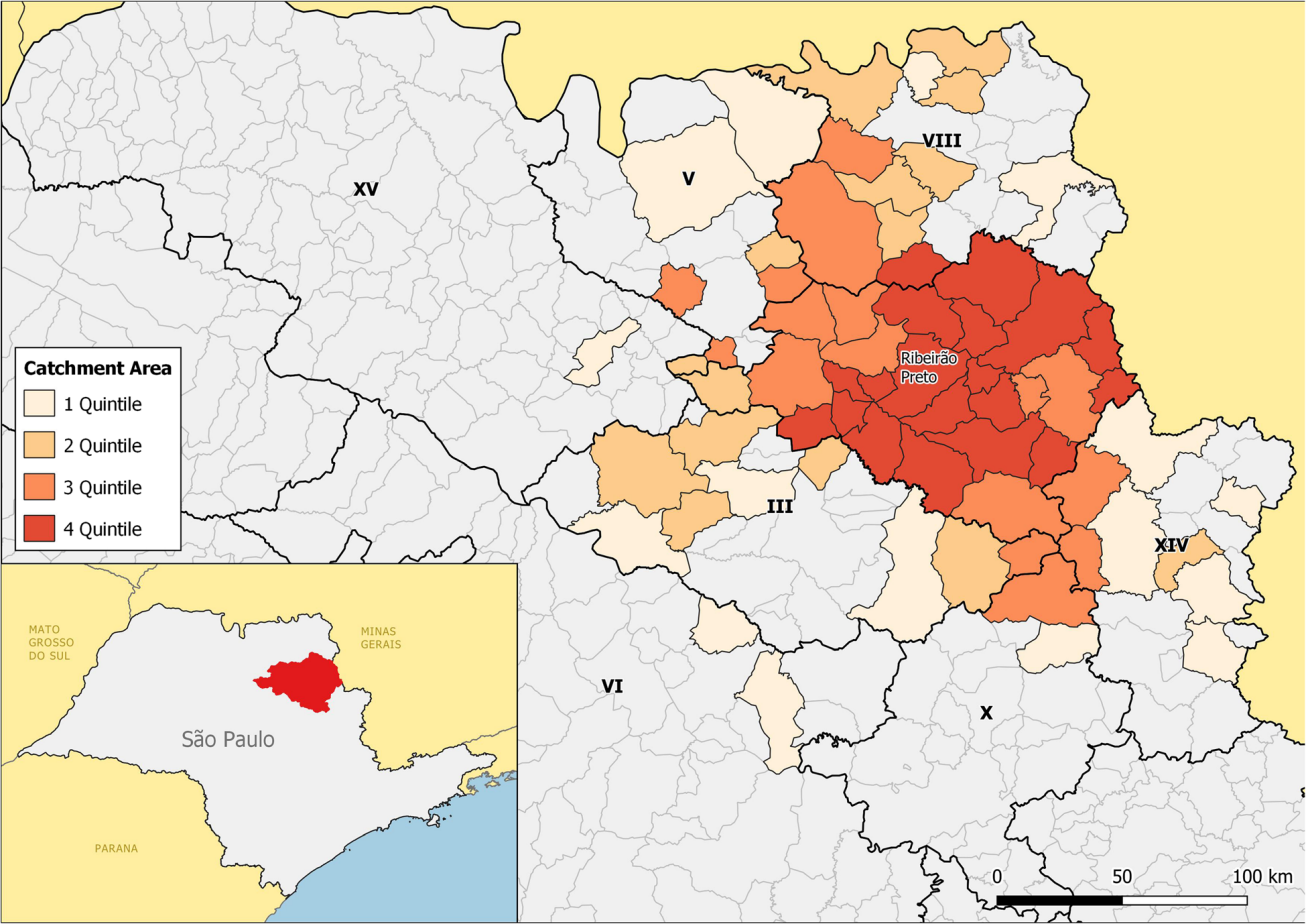
Catchment area of municipalities with hospitalization, by quartile of intensity, 2012. Legends: Intensity of colors expresses the proportion of flows in quartiles. Source: [28, 29]

For a more specific analysis of this region, we compared the density of migration towards the main attracting municipalities, with the expected number of births in each city of origin of the patients, calculated using the birth rate of DRS XIII for the Census year 2010 (Fig. 3).

The map shows that the functional regions surrounding those cities performing deliveries show geographic proximity gradients but not necessarily territorial continuity, due to the connectivity of highways, bus lines, and other factors. The highway system in the São Paulo state is a well-developed network, and most of these municipalities are within a 90-min range from Ribeirão Preto, but we were unable to measure real times of displacement in this research. In any case, the crucial influence of highway connections and mass transportation should be factored in the design of regions. This may be important because it questions one of the presuppositions in any regionalization scheme, the contiguity of the territorial units.

To measure the sufficiency of municipalities for resolution of pregnancy, we compared the inflow and outflow of patients, compared to the total number of patients with residence in the city. The municipality of Ribeirão Preto, is an “exporter” of services (therefore “importer” of patients) as it has a LOFI greater than 90%, but a LIFO less than 75%, which means that it is sufficient for its residents, but it needs to be integrated within a larger region from which it “imports” patients. Conversely, the municipality of Pontal that has a LOFI of 66% and a LIFO of 100% (it does not receive patients from outside and only 66% of its patients are admitted in the municipality) is not considered sufficient because it needs to be integrated into a wider region to solve 34% of the cases of its population (Table 1).

**Table 1:** Hospital sufficiency for pregnancy, childbirth, and puerperium. Municipalities with hospitalization, micro-regions, and DRS XIII—2012

	Little in from outside (LIFO)	Little out from inside (LOFI)	Sufficiency level LIFO and LOFI > 75%
Altinópolis (VdC)	98%	81%	Sufficient
Cajuru (VdcC)	88%	93%	Sufficient
Cravinhos (AG)	100%	0%	Insufficient
Guariba (HV)	86%	77%	Sufficient
Jaboticabal (HV)	74%	89%	Insufficient
Monte Alto (HV)	100%	81%	Sufficient
Pontal (HV)	100%	66%	Insufficient
Ribeirão Preto (AG)	71%	100%	Insufficient
Santa Rosa de Viterbo (AG)	100%	37%	Insufficient
São Simão (AG)	100%	28%	Insufficient
Serrana (AG)	87%	75%	Sufficient
Sertãozinho (HV)	77%	95%	Sufficient
Micro-region VdC	93%	73%	Insufficient
Micro-region HV	71%	80%	Insufficient
Micro-region AG	84%	72%	Insufficient
Overall DRS XIII	78%	96%	Sufficient

**Fig. 4:**
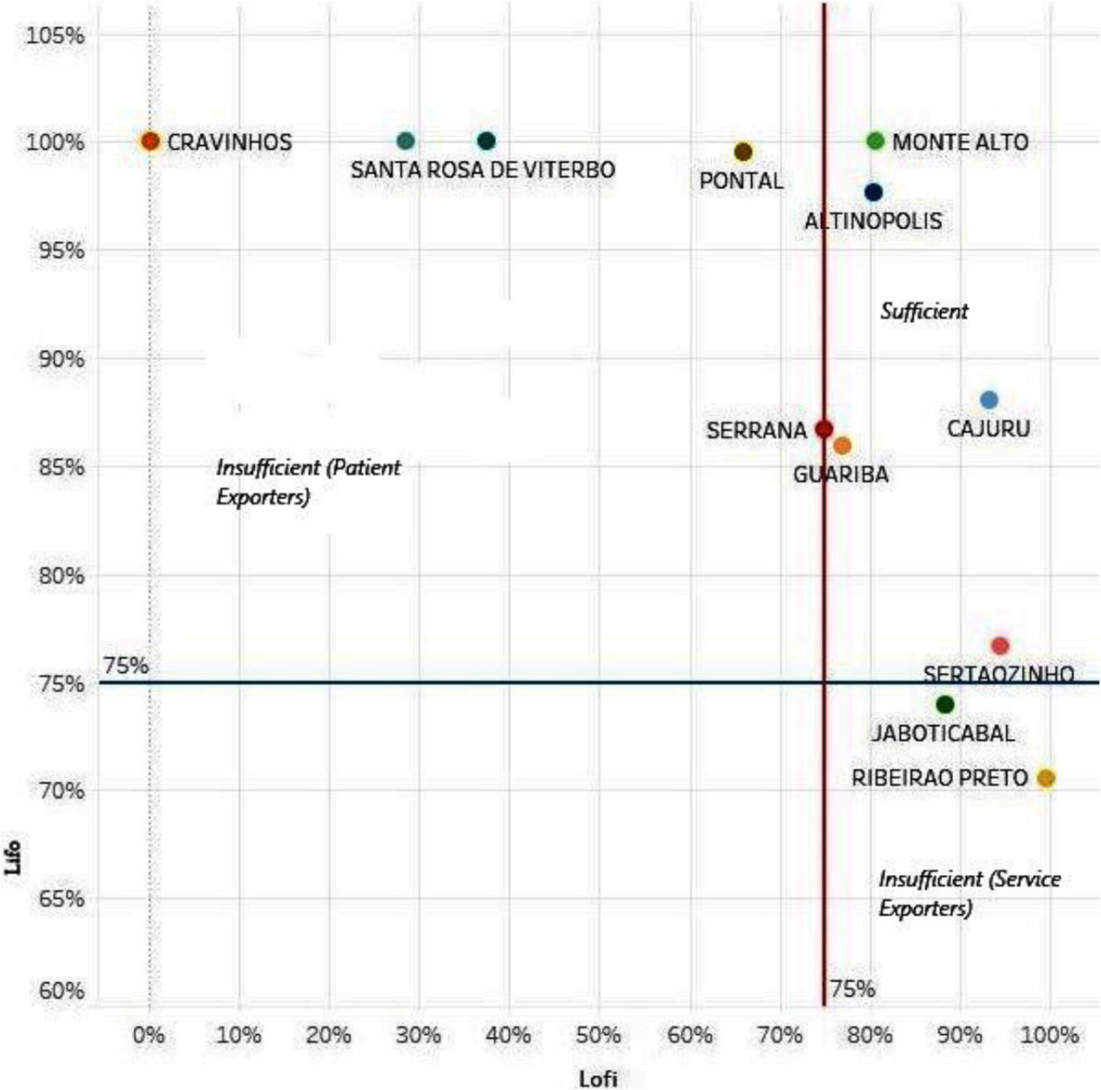
Municipal sufficiency rate Lofi > 75% and Lifo > 75% of the patients for pregnancy, delivery, and puerperium. Source: [42, 43]

When using the successive enlargement of areas proposed by Frech et al. [42], it was possible to test the sufficiency or independence of the three DRS XIII micro-regions. None of these micro-regions reached the LOFI/LIFO sufficiency level. Finally, we tested the complete set of the three micro regions together, resulting in a LIFO of 78% and LOFI reaching 96%, consistent with the panorama of a DRS globally sufficient and with a tendency to “import” patients from outside (Table 1 and Fig. 4).

For the purposes of network analysis of cross-border flows, we used the concept first developed by Taliaferro and Remmers [43], considering that each displacement of a patient from the municipality *i* (residence) to the municipality *j* (hospitalization) constitutes a directed (i.e., from the residence to the hospital) edge or arc between two vertices. The sum of the arcs between two vertices *i* and *j* is equal to the number of patients who moved from municipality *i* to municipality *j* to receive hospitalization in the year 2012.

**Fig. 5:**
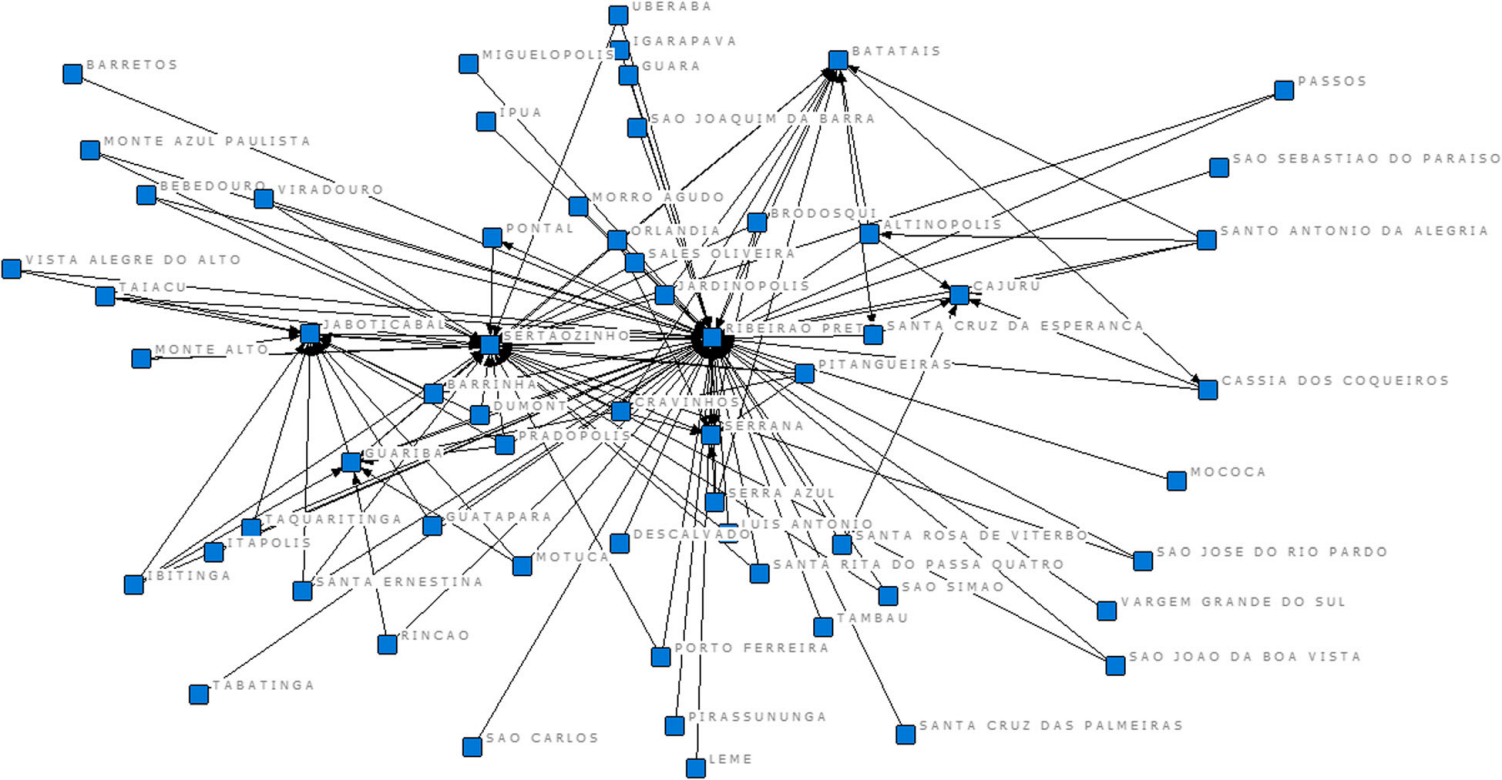
Network of hospitalization flows due to pregnancy, childbirth, and puerperium in DRS XIII and other municipalities

In the representation of the network, we can see the intense set of flows within the region as well as the existence of a central core in Ribeirão Preto, and smaller ones in Sertãozinho, Jaboticabal, and Cajuru (Fig. 5).

**Fig. 6:**
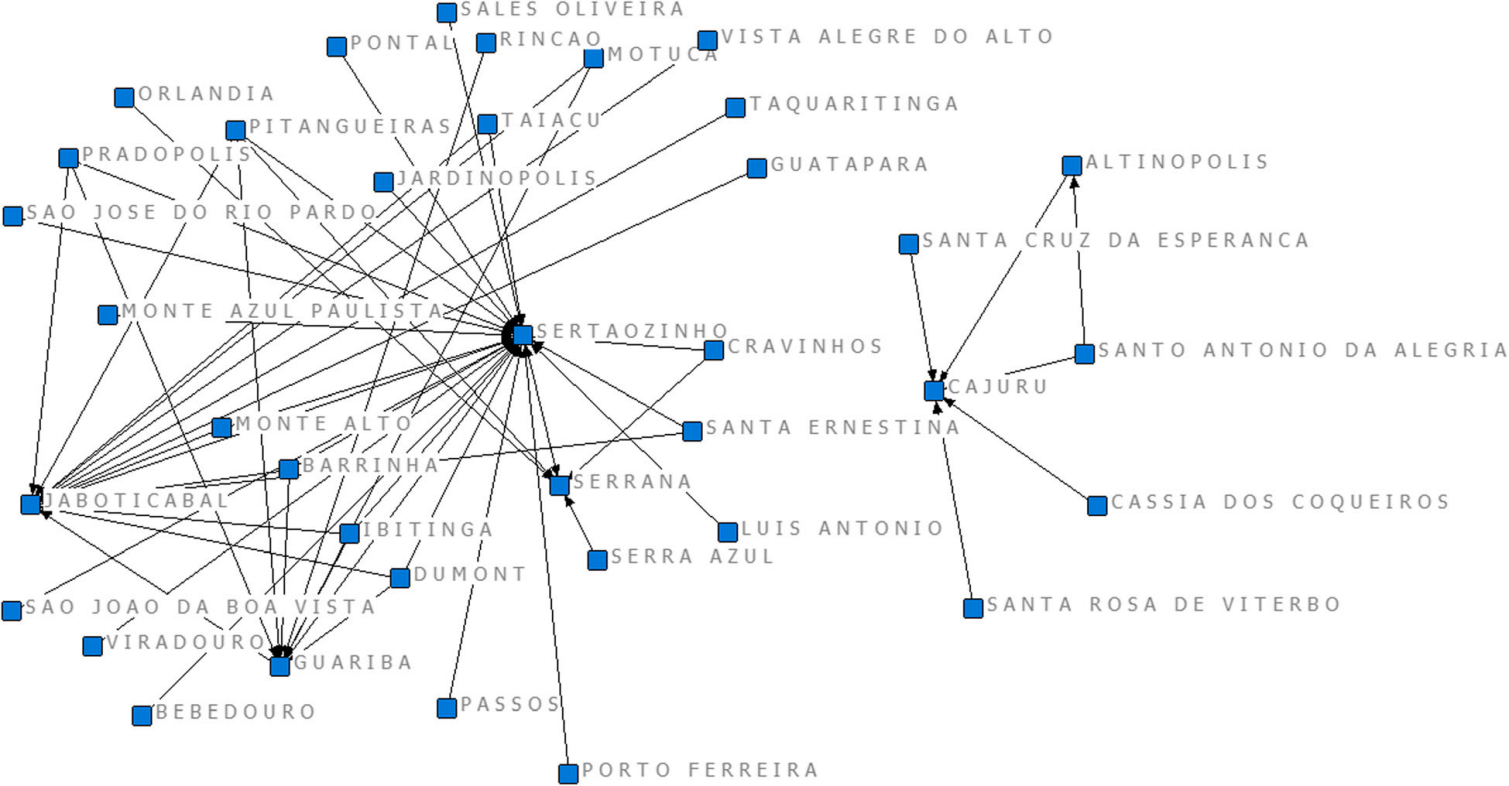
Network of hospital admissions for pregnancy, childbirth, and puerperium in DRS XIII and other municipalities, excluding Ribeirão Preto

Due to its important centrality, Ribeirão Preto was excluded in a second stage of the analysis, in order to examine the remaining network structure. This allowed having a clearer picture of the connections of the peripheral clusters that offer services to surrounding regions. Figure 6 shows that there is a Cajuru-centered network that does not connect with the rest, of small size, being relevant for the flow of patients from only five municipalities.

## Discussion

The results depict the DRS XIII patients’ movements for the resolution of their pregnancy in the region surrounding Ribeirão Preto. This set of displacements configures patterns of the agents’ behavior, due to the interdependence of the municipalities to fulfill the different demands for delivery-related hospitalizations. Looking at these patients’ movements using the lens of complexity, they can be interpreted as emergent behaviors limited by the regional administrative boundaries and other norms that constitute top-down constraints [3].

Several patterns of emergent behaviors may be analyzed. More than 25% of the admissions related to childbirth come from patients crossing borders to a different municipality to be hospitalized. The functional region found in the study is broader than the limits established by the ordinances and provisions of the State Department of Health, due to the strong influence of Ribeirão Preto to attract patients. There is a mismatch between this data-derived region and the boundaries established in the legal division of the state. A recent study in Turkey found that 22% of the Ministry of Health region boundaries did not match the regions emerging from patient mobility [44]. The study of patients’ flows to hospitals in an Australian state, in order to determine the hospital service area networks (HSAN) showed that 30% of patients came from outside of the designated HSAN areas [45]. The constraints posed by the political and administrative divisions of geographical regions are barriers to the capacity of self-organization that communities may achieve using their own emergent patterns of use as guidance.

Municipalities that are not fully contiguous compose the catchment area surrounding the hospitals. This may be important because it questions contiguity as one of the presuppositions in any regionalization scheme, and is likewise being observed regarding urban areas as in the proposed zoning along main transit axes in the city of São Paulo [46] and in the rural/urban mix around small rivers in the Amazon basin [47], calling for a multi-scale approach [48].

Patients and practitioners show a preference to refer cases to be hospitalized in Ribeirão Preto due to the higher level of its facilities and staff and the availability of resources. This preference may be perceived in the fact that a significant number of pregnant women go to the hospitals of Ribeirão Preto for low-risk procedures. Another indicator of this preference for Ribeirão Preto in the case of birth-related events is the higher willingness to travel of these patients, showing displacements twice as long as patients who receive care in other municipalities, concurring with what is observed in other contexts [26, 49].

Regulatory mechanisms contribute to these preferential flows by the administrative rule of using the best available hierarchy in terms of service, thus sending the patients to the services of Ribeirão Preto and specifically to the HC-FMRPUSP, reinforcing the centrality of this municipality. These accumulated evidences point to the effect of the preferences of the agents (patients, physicians, regulators) in the direction of the flows.

A general landscape of the flows in the region is dominated by the sufficiency of DRS XIII as a whole (due to Ribeirão Preto’s strong “exporter of services” profile). Fukuoka et al. [33] using data from 2007 to 2008 showed the sufficiency to resolve pregnancy-related hospitalizations in several cities of the DRS XIII. The present study shows that this municipal sufficiency is contrasted by the fact that none of the three micro-regions achieves enough autonomy, showing the need for further sub-regional consolidation. Alves [31] proposed the use of the concept and metrics of entropy (disorder) to measure the regulation of the flows of patients needing admissions in other municipalities. The entropy index for origin is low when a maximum of residents of one municipality move to be treated exclusively in one hospital; at the opposite, the index is high when there is a wider variation in points of destination for hospitalization. The aforementioned study found that the change from four to three micro-regions in DRS XIII in 2007 was linked to a greater order (expressed as lower entropy) of the flows that presented improved coherence between places of origin and destination in the new regional design.

The successive and non-fully successful alternatives of regionalization and distribution of services in the territories [14, 43] shed light over the aforementioned wicked problem related to the optimal way of combining the best technical quality with the greatest accessibility for the different types of services that parturients can demand. In a universal health system as is in the Brazilian case, this issue involves combining several partially contradictory approaches: a logistic approach based on the problems generally called “the traveling salesman,” trying to minimize the displacements (and their costs); a technical quality approach, seeking to maximize the deployment of high-quality services under the restrictions of resources in most Brazilian municipalities; and an agents’ preference approach with a focus on patients’ individual preferences, who have the right to choose how and where to have their baby.

The logistic approach [49, 50] shows the contradiction between having very specialized centers, well-equipped and trained, inevitably scarce, and therefore less accessible, versus multiple services widely distributed in the territory, albeit presenting compromised technical quality due to human and equipment insufficiencies. In this aspect, there are studies demonstrating that the clinical results depend on the technical experience of teams and equipment regarding the procedures in question [21, 22, 51, 52]. Studies in contexts as diverse as the regional distribution of angioplasty in Italy [53], the referral of patients to hospitals in various regionalization layouts in Canada [54], and rural patients in Tanzania [55] all show the so-called severity effect. When there is a perception of potentially life-threatening situations, the distance to the treatment site has a lesser effect as a perceived impediment by patients, who prefer to travel in order to achieve better quality treatment. This effect is related to the results of the present study, observing that patients prefer to travel greater distances for the resolution of pregnancy in Ribeirão Preto, even in cases of low risk.

The logistic approach is mediated and modified by the technical quality expected for a given service**.** The distinction made by Kongnyuy et al. [56] between Basic Obstetric Care and Comprehensive Obstetric Care can help to find a technical quality parameter. Basic obstetric care includes procedures that provide for safe simple deliveries, while comprehensive obstetric care adds the ability to perform cesarean sections and blood transfusion service. The five major causes of maternal mortality in developing countries (which together account for 99% of maternal mortality in the world) are hemorrhage, septicemia, unsafe abortion, eclampsia, and obstructed labor [57, 58]. Therefore, a good answer to the dilemma of the regional distribution of childbirth services may be that they should be widely distributed as long as they can certify that they provide comprehensive care according to the Kongnyuy et al. [56] definition. The present study should be used as a basis for an expanded mapping of the hospitals equipped with the abovementioned capacities in order to match the regional flows of needs with the deployment of resources able to respond to those needs.

The topic of agent preferences in selecting the place of resolution of pregnancy has raised global interest, motivating studies in both rich and poor countries for different reasons. In rich countries, these studies are motivated by the humanization movements and the empowerment of women’s decisions regarding childbirth [59–61]. On the other hand, in poor countries, the studies are oriented to understand the motivations of pregnant women and their families to define the type and place of care in order to promote deliveries in health centers well equipped for obstetric care [55, 62–64].

If considered jointly, the set of abovementioned studies helps to understand the observed dynamics in the present study, in order to use patients’ flows as a guiding element for reorganization of the health system. Using a complexity approach, it proposes several ways to characterize the emergent patterns of these flows. The examination of the flows should be considered as expression of how patients and providers are agents for adaptation of the administrative constraints. At the same time, it is possible to draw consequences from the flows as bottom-up guiding principles for reshaping the health systems constraints and improving the adequacy of availability of services to the population needs [3].

The complex systems analysis proposed by Vandenbroeck et al. [65] may be useful to understand the patient flows for the resolution of pregnancy, childbirth, and puerperium as emerging from the convergence of several sub-systems. Using Vandenbroeck terms, we can characterize four “engines” that operate separately, with multiple feedback circuits among them.

A first engine is the demo-epidemiology, the dynamics of populations in their settlements, and the health conditions (each of these factors in itself a complex system). As seen in this study, the demographic and epidemiological conditions of the DRS XIII and the surrounding region are relatively homogeneous and therefore not conducing to irregular flows, as could be the case if there were extremely poor or overpopulated municipalities. The second engine encompasses the facilities’ deployment with its own dynamics, related to technological developments, the economic factors related to investments in health and management models and funding (also in each case, complex systems with their own dynamics). In the case of the region studied, several municipalities have hospitals that are supposedly capable of performing deliveries, but at the same time the technological and evolutionary dynamics of what WHO calls comprehensive obstetric care show that some of them may not have the ability for events that exceed basic obstetric care. This “engine” therefore influences flows towards higher-level hospitals and increases patients’ flows.

The third engine is the political geography of the region, encompassing territories, their political organization, the dynamics of regionalization, and communications (which modulate distances). As already seen, DRS XIII has mechanisms of flow regulation and should promote resource pooling. However, as shown in discussions that came to the public in July 2015 [66], municipal stakeholders do not accept the possibility of combining resources and contributing to maintain centers outside their own limits that would otherwise allow the sufficiency of the micro-regions. Thus, a focus on distance or the simple provision of transport or the residence in a municipality may reveal little about patients’ willingness to travel for health care as one element in their decisions about the choices offered [26, 67].

Finally, the fourth engine consists of the preferences of the agents, in the present case, the decisions of the pregnant women, families, physicians, and other providers of health services shown to be important due to the influence in patients’ flows that do not appear to have technical justification.

The study suffered from several limitations: the patients’ flow due to procedures related to pregnancy, delivery, or puerperium was assumed whenever the municipality of residence and the municipality of hospitalization were different. This is not always the case, as some of the patients may have moved for other reasons and had not changed their registered residence. A second limitation is due to the fact that even in the most complete versions of the database (2012), there are hospitals that do not show complete data, and the database captures the patients that are hospitalized within DRS XIII; therefore, those patients residing in DRS XIII admitted to hospitals in other regions of the state or in other states are not included. A third limitation has to do with the distances traveled, as the study used the distances from the centroids of municipalities, due to the lack of postal codes on the database. Therefore, true origin-destination distance is not computed, as the municipalities in the studied area measure an average of 360 km^2^ and an average radius from the centroid of 10.7 km.

## Conclusion

The study of emergent patterns from patient flows within a region and their use as guiding elements in modifying the regional configuration of a health system has three main angles that should be noted. Firstly, the study highlights the many dimensions that a universal health system should pay attention to make effectively accessible the kind of healthcare postulated by the law and regulations, including the behavior of the agents as key drivers in the system design. Secondly, it calls the attention to the need of avoiding simplistic views that propose that an isolated element or a specific intervention (geographic-based regional division, regulation of access, opening or closing of maternity wards) will solve or permanently modify a complex web of interactions. The study shows that apprehending the multiple system dynamics requires a different approach in order to guide more adequate interventions. The output of this research may be used as comparison in order to map out the present regional distribution of childbirth services and their capacity to provide comprehensive care. As a result, it may help to match the regional flows of needs with the deployment of resources able to respond in an effective, efficient, and equitable way to the obstetric needs. Finally, the study has potential to be applied to map out other type of patient flows, such as cancer or specialized surgery probably suggesting the need of multi-level regionalization designs in order to reorient the functioning of the Brazilian Health System through a patient-centered approach.

## Data Availability

A spreadsheet with the full matrix on which the network analysis was performed is available upon request from the corresponding author.

## Abbreviations

DGO-FMRPUSP: Department of Gynecology and Obstetrics of the Ribeirão Preto Medical School, University of São Paulo
DRS XIII: Regional Health Department XIII, State of São Paulo, Brazil
HSAN: Hospital Service Area Network
LIFO: Little in from outside
LOFI: Little out from inside
ORAH: Regional Observatory for Hospital Care
SUS: Brazilian Universal Health System
WHO: World Health Organization
